# RNA Sequencing and Coexpression Analysis Reveal Key Genes Involved in α-Linolenic Acid Biosynthesis in *Perilla* *frutescens* Seed

**DOI:** 10.3390/ijms18112433

**Published:** 2017-11-16

**Authors:** Tianyuan Zhang, Chi Song, Li Song, Zhiwei Shang, Sen Yang, Dong Zhang, Wei Sun, Qi Shen, Degang Zhao

**Affiliations:** 1 Rapeseed Research Institute, Guizhou Academy of Agricultural Sciences, Guiyang 550008, China; zhangtianyuan@foxmail.com (T.Z.); szw1225@163.com (Z.S.); yangsen.90@163.com (S.Y.); 2 The Key Laboratory of Plant Resources Conservation and Germplasm Innovation in Mountainous Region (Ministry of Education), Guizhou University, Guiyang 550025, China; lpsssl@126.com; 3 Institute of Chinese Materia Medica, China Academy of Chinese Medical Sciences, Beijing 100700, China; Csong@icmm.ac.cn (C.S.); dzhang1987@icmm.ac.cn (D.Z.); wsun@icmm.ac.cn (W.S.)

**Keywords:** *Perilla frutescens*, RNA sequencing (RNA-seq), α-linolenic acid (ALA), triacylglycerol (TAG) biosynthesis, herbgenomics

## Abstract

*Perilla frutescen* is used as traditional food and medicine in East Asia. Its seeds contain high levels of α-linolenic acid (ALA), which is important for health, but is scarce in our daily meals. Previous reports on RNA-seq of perilla seed had identified fatty acid (FA) and triacylglycerol (TAG) synthesis genes, but the underlying mechanism of ALA biosynthesis and its regulation still need to be further explored. So we conducted Illumina RNA-sequencing in seven temporal developmental stages of perilla seeds. Sequencing generated a total of 127 million clean reads, containing 15.88 Gb of valid data. The de novo assembly of sequence reads yielded 64,156 unigenes with an average length of 777 bp. A total of 39,760 unigenes were annotated and 11,693 unigenes were found to be differentially expressed in all samples. According to Kyoto Encyclopedia of Genes and Genomes (KEGG) pathway analysis, 486 unigenes were annotated in the “lipid metabolism” pathway. Of these, 150 unigenes were found to be involved in fatty acid (FA) biosynthesis and triacylglycerol (TAG) assembly in perilla seeds. A coexpression analysis showed that a total of 104 genes were highly coexpressed (*r* > 0.95). The coexpression network could be divided into two main subnetworks showing over expression in the medium or earlier and late phases, respectively. In order to identify the putative regulatory genes, a transcription factor (TF) analysis was performed. This led to the identification of 45 gene families, mainly including the AP2-EREBP, bHLH, MYB, and NAC families, etc. After coexpression analysis of TFs with highly expression of FAD2 and FAD3 genes, 162 TFs were found to be significantly associated with two FAD genes (*r* > 0.95). Those TFs were predicted to be the key regulatory factors in ALA biosynthesis in perilla seed. The qRT-PCR analysis also verified the relevance of expression pattern between two FAD genes and partial candidate TFs. Although it has been reported that some TFs are involved in seed development, more direct evidence is still needed to verify their function. However, these findings can provide clues to reveal the possible molecular mechanisms of ALA biosynthesis and its regulation in perilla seed.

## 1. Introduction

*Perilla frutescens*, a traditional food and medicinal plant, belongs to the family Lamiaceae [1]. It is widely cultivated in East Asian countries, especially in China, Korea, and Japan. There are two cultivated varieties of perilla—*Pf.* var. *frutescens*, mainly used for edible and health purposes, and *Pf.* var. *crispa,* mainly used as medicine, vegetable, and spice [2]. The seeds of *Pf.* var. *frutescens* contain approximately 45–55% oil, which comprises 54–64% α-linolenic acid(ALA) (C18:3) [3]. The ALA content of perilla seeds is significantly higher than that of the other major oil seed crops, e.g., soybean (5–13%) [4], rapeseed (1–8%), maize (0.5–2%), and sunflower (0.5–2%) [5]. Linolenic acid (C18:3) plays an important role in the maintenance of brain nerve system and has an obvious positive effect on intelligence, memory, and eyesight, but is usually scarce in our daily meals [6,7,8]. Therefore, the oils of perilla seed have attracted wide attention in research and health domain. However, the mechanism of ALA and its regulation in perilla remains unclear.

With the development of high-throughput sequencing technology, RNA-seq has become an effective method to analyze the spatiotemporal expression of a gene and to obtain more comprehensive information about gene transcription and regulation [9]. Some oil crop plant seed contain specific fatty acid (FA) contents. Understanding those specific lipid formation process is extremely important to unravel the unique lipid metabolism and regulation mechanisms [10]. Recently, transcriptome sequencing and characterization based on the Illumina second-generation sequencing technology has enabled the rapid identification and profiling of mechanisms involving oil content and FA composition in various oil plants, such as soybean [11,12], peanut [13,14], and palm oil [15]. Similarly with perilla, the flax [16], sea buckthorn [17], *Camelina sativa* [18], *Plukenetia volubilis* L. [19], and tree peony [20] also rich in ALA in seed oil. In sequencing of flax seed, the key embryogenesis regulators were mined [14]. And more biosynthesis genes contributing in ALA accumulation have been identified in those plant seeds [11,12,13,14,15,16,17,18,19,20]. For perilla, 540 unique genes involved in acyl-lipid metabolism from four developmental stages in perilla seeds, and characterized the expression profiles of 43 genes involved in FA and triacylglycerol (TAG) synthesis [21].

Coexpression analysis, based on the “guilt-by-association” principle, is used to identify the genes that have similar expression patterns and are more likely to be functionally associated [22]. Recently, coexpression analysis has proved to be a powerful tool for identifying genes and regulatory factors in transcriptional networks in human [23,24], plants [25,26,27,28], and animals [29,30]. In *Arabidopsis*, a large number of transcription factors (TFs) involved in seed development, FA and protein biosynthesis, flavone metabolism, and several important metabolic pathways were identified by using the coexpression method [31,32,33,34].

In this study, we conducted an Illumina RNA sequencing in seven developmental stages of perilla seeds. The coexpression of lipid metabolism genes and TFs identification provided more genes and regulation involving in ALA biosynthesis. Overall, the transcriptome-wide identification and coexpression analysis provide a foundation for possible molecular mechanisms of ALA biosynthesis and its regulation in perilla seed.

## 2. Results

### 2.1. Transcriptome Sequencing and Assembly

**Figure 1:**
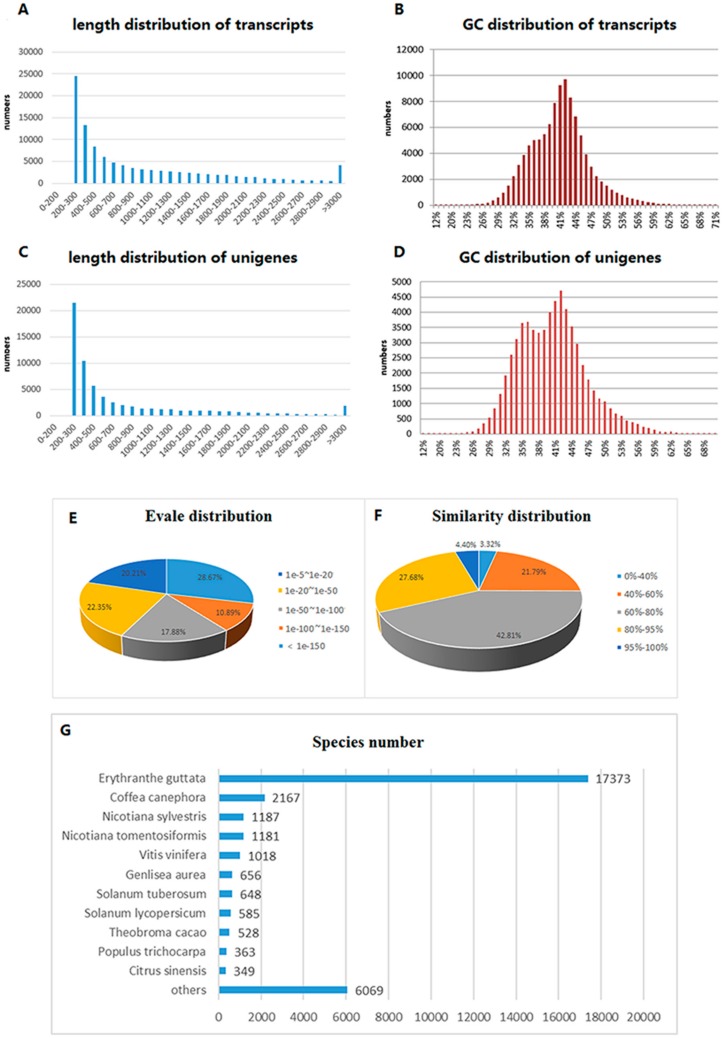
Overview of the de novo assembly of transcriptome sequencing in *Perilla frutescens* and annotation based on a non-redundant (NR) protein database. Length (**A**) and GC distribution (**B**) of transcripts; length (**C**) and GC distribution (**D**) of unigenes are shown; (**E**) *e*-value distribution of BLAST hits for the assembled unigenes; (**F**) Similarity score distribution of the top BLAST hits for the assembled unigenes; (**G**) Species distribution of the top BLAST hits for the assembled unigenes.

In order to explore the possible molecular mechanism of ALA biosynthesis and its regulatory in perilla seed, seven different seed developmental stages after flowering (2, 6, 10, 14, 18, 22, and 26 DAF) were collected and subjected to Illumina paired-end sequencing. After removing adaptor sequences and filtering low-quality and ambiguous reads, a total of 127 million clean reads, containing 15.88 Gb of valid data were acquired. All sequencing statistics are shown in Table 1. Subsequently, we obtained 104,638 transcripts and 64,156 unigenes by de novo assembly using the Trinity program (Table 2). The average length of transcripts was 968 bp and that of N50 was 1608 bp. Furthermore, the average length of unigenes was 777 bp and that of N50 was 1417 bp. Of all unigenes, 20,965 (32.68%) possibly contained complete open reading frames (ORFs), which were predicted by the program TransDecoder. The GC content and length distribution of all assembled unigenes and contigs are presented in Figure 1. The sequencing data have been deposited in the National Center for Biotechnology Information (NCBI) database (Accession number: SRP111892).

### 2.2. Functional Annotation and Classification

For functional annotation, all assembled unigenes were searched against the a non-redundant (NR), Swiss-Prot, TrEMBL, and Clusters of Orthologous Groups (COG), Gene Ontology (GO), and Kyoto Encyclopedia of Genes and Genomes (KEGG) databases (Table 3).

In NR database, a total of 32,132 (50.08%) unigenes showed matched items. Among them, 9211 (28.67%) unigenes showed an *e*-value less than 1e^−150^, and 3500 (10.89%) unigenes had an *e*-value between 1e^−100^, and 1e^−150^ (Figure 1E). In addition, 1413 (4.40%) unigenes exhibited alignment identities greater than 95%, and 8,895 (27.68%) exhibited alignment identities between 80% and 95% (Figure 1F).

The species annotation results showed that 17,373 (54.08%) unigenes were highly matched with *Erythranthe guttata*. Other unigenes matched with *Coffea canephora* (6.75%), *Nicotiana sylvestris* (3.70%), *Nicotiana tomentosiformis* (3.68%), and *Vitis vinifera* (3.17%) (Figure 1G).

However, only 86 unigenes matched with *P. frutescens* sequences in the NR database. It is possible that the genomic and transcriptomic information is currently lacking for perilla in the NR database (only 105 sequences).

Based on the alignment of paralogs or orthologs, 8654 unique sequences matched in the COG database were clustered into 25 functional categories (Figure S1). Among the 25 categories, the largest category was general function prediction only (2702; 31.22%), followed by transcription (1727; 29.96%) and replication, recombination and repair (1652; 19.09%). A GO functional classification was used to classify unigene functions on the basis of NR annotation. Furthermore, 22,263 (34.70%) unigenes were assigned to one or more GO terms, which contained cellular component (18,113; 81.36%), molecular function (18,847; 84.66%), and biological process (18,119; 81.39%) (Figure S2).

A KEGG pathway analysis was performed to identify the biological metabolism pathways. A total of 10,904 unigenes were grouped into 301 KEGG pathways, mainly including the organismal systems (1661), metabolism (4818), genetic information processes (2479), environmental information processing (1098), and cellular processes (1133) (Figure S3).

### 2.3. Genes Related to Lipid Biosynthesis in Perilla Seed

Based on the annotation results, 371 (4.01%) unigenes in COG annotation were assigned to lipid transport and metabolism, 2624 (11.79%) unigenes were assigned to the GO term “developmental process,” and 486 (4.3%) unigenes in the KEGG annotation were assigned to lipid metabolism pathway, mainly including “fatty acid degradation” (67 unigenes; ko00071), “glycerolipid metabolism pathway” (78 unigenes; ko00561), “biosynthesis of unsaturated fatty acids” (42 unigenes; ko01040), “linoleic acid metabolism pathway” (21 unigenes; ko00591), and “α-linolenic acid metabolism pathway” (64 unigenes; ko00592) (Table S1).

**Figure 2:**
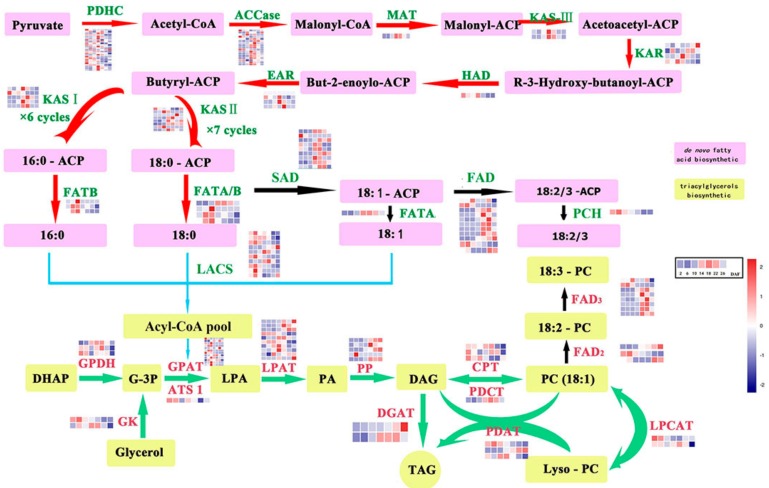
Perilla sequences associated with fatty acid biosynthetic pathway. Each row represents a gene, and each column represents a specimen (stage). Depths of color in the red and blue rectangles indicate higher and lower represents the Z-score RNA expression lever. Identified enzymes include: PDHC: pyruvate dehydrogenase complex; ACCase: acetyl-CoA carboxylase; MAT: malonyl-CoA ACP transacylase; ACP: acyl carrier protein; KAS I, II, III: ketoacyl-ACP synthase I, II, III; KAR: ketoacyl-ACP reductase; HAD: hydroxyacyl-ACP dehydrase; EAR: enoyl-ACP reductase; SAD: stearoyl-ACP desaturase; FAD: fatty acid desaturase; FATA/B: fatty acyl-ACP thioesterase A/B; PCH: palmitoyl-CoA hydrolase; LACS: long-chain acyl-CoA synthetase; FAD2: oleate desaturase (endoplasmic reticulum); FAD3: linoleate desaturase; GK: glycerol kinase: GPDH: glycerol-3-phosphate dehydrogenase; GPAT: glycerol-3-phosphate acyltransferase; LPAT: 1-acylglycerol-3-phosphate acyltransferase; ATS1: glycerol-3-phosphate O-acyltransferase; PP: phosphatidate phosphatase LPIN; DGAT, acyl-CoA: diacylglycerolacyltransferase; PDAT: phospholipid:diacylglycerol acyltransferase; LPCAT: 1-acylglycerol-3-phosphocholine acyltransferase; CPT: diacylglycerol cholinephosphotransferase; PDCT: phosphatidylcholine:diacylglycerol cholinephosphotransferase.

For acquiring more knowledge on FA biosynthesis and TAG assembly pathway genes in perilla seed, more attention was paid to the 150 unigenes that were annotated as FA biosynthesis and TAG assembly genes (Figure 2 and Table S2). In this study, we found 40 FA biosynthesis unigenes, of which 25 encoded pyruvate dehydrogenase complex (pdh A, B, C, and D) subunits, 14 encoded acetyl-CoA carboxylase (ACCase) subunits, and one encoded malonyl-CoA ACP transacylase (MAT). A total of 39 unigenes were involved in fatty acid acyl chain elongation; of these, 15 unigenes encoded 3-ketoacyl-ACP synthases (KAS), one unigene encoded hydroxyacyl-ACP Dehydrase (HAD), four unigenes encoded ketoacyl-ACP reductase (KAR), four unigenes encoded enoyl-ACP reductase (EAR), five unigenes encoded acyl-ACP thioesterase (FAT) and palmitoyl-CoA hydrolase (PCH), and 10 unigenes encoded long-chain acyl-CoA synthetases (LACS). Of the 20 unigenes involved in fatty acid desaturase, nine encoded stearoyl-ACP desaturase (SAD), three encoded oleate desaturase (FAD2), and eight unigenes encoded linoleate desaturase (FAD3).

The Kennedy pathway in perilla seed showed the involvement of 36 unigenes in the TAG assembly pathway. Of these unigenes, three encoded glycerol-3-phosphate dehydrogenase (GPDH), 13 encoded glycerol-3-phosphate acyltransferase (GPAT) and homologous gene (glycerol-3-phosphate-*O*-acyltransferase ) ATS1, two encoded glycerol kinase (GK), 10 encoded lysophosphatidic acid acyltransferase (LPAT) and homologous gene phospholipase A2 (PLA2), five encoded phosphatidic acid phosphatase (PAP). Two unigenes encoded acyl-CoA: diacylglycerol acyltransferase (DGAT, which transfers an acyl group from acyl-CoA to the sn-3 position of sn-1,2-diacylglycerol to form TAG). Three unigenes encoded phospholipid: diacylglycerolacyltransferase (PDAT), which preferentially transfers an acyl group from the sn-2 position of a phospholipid to diacylglycerol. A unigene encoding phosphatidylcholine: diacylglycerol choline phosphotransferase (PDCT) and three unigenes encoding diacylglycerol cholinephosphotransferase (CPT) were identified in the perilla seed.

### 2.4. Differentially Expressed Genes (DEGs)

To identify the DEGs in a developed seed of perilla, we calculated the FPKM (Fragments Per Kilobase of exon per Million mapped fragments) values of the assembled unigenes at each developmental stage of perilla seed and performed the pairwise comparison of different development stages. A total of 17,416 unigenes were found to be widely expressed in each samples (FPKM > 1 in all samples). Among them 11,693 unigenes showed more than two-fold different expression level. Among samples, the 6DAF vs. 10DAF had 294 up-regulated and 558 down-regulated unigenes, and the 10DAF vs. 14DAF had 353 up-regulated and 368 down-regulated unigenes, which were the two most highly different adjacent stages (Table 4).

Subsequently, we performed GO analyses to identify the function of these DEGs (Figure S2). In the GO classification analysis, 7501 unigenes were assigned to three main GO functional categories. The largest sub-category in the “molecular function” category was ion binding (2928 unigenes, accounting for 25.04% of all DEGs), followed by DNA binding (911, 7.62%), and oxidoreductase activity (878; 7.51%). In “cellular component”, the largest sub-category was cell (5237; 44.79%), followed by intracellular (4363; 58.17%), and organelle (3792; 37.31%). In “biological process”, the largest sub-category was biosynthetic process (2214; 18.93%), followed by cellular nitrogen compound metabolic process (1594; 13.63%), and response to stress (1275; 10.90%).

### 2.5. Coexpression of Lipid Metabolism Genes

The transcriptome-wide coexpression analysis showed that out of the 486 perilla lipid metabolism genes, 104 were highly correlated (*r* > 0.95). The coexpression network presenting two main links (containing 89 unigenes) could be divided into two relatively independent subnetworks (I and II). Subnetwork I contained 35 unigenes, mainly including the TAG biosynthesis genes, such as SAD, LACS, PP, LPAT, and DGAT. Whereas subnetwork II contained 57 unigenes, mainly including the de novo FA synthesis genes, such as ACCase, PDCH, KAS, SAD, and FATA, and some transferase and peroxidase. Fifteen unigenes, mainly including aldehyde dehydrogenase (NAD+, ALDH), ceramide kinase (CKER), ceramide synthetase (CERS), and so on, showed relatively less correlation with others.

**Figure 3:**
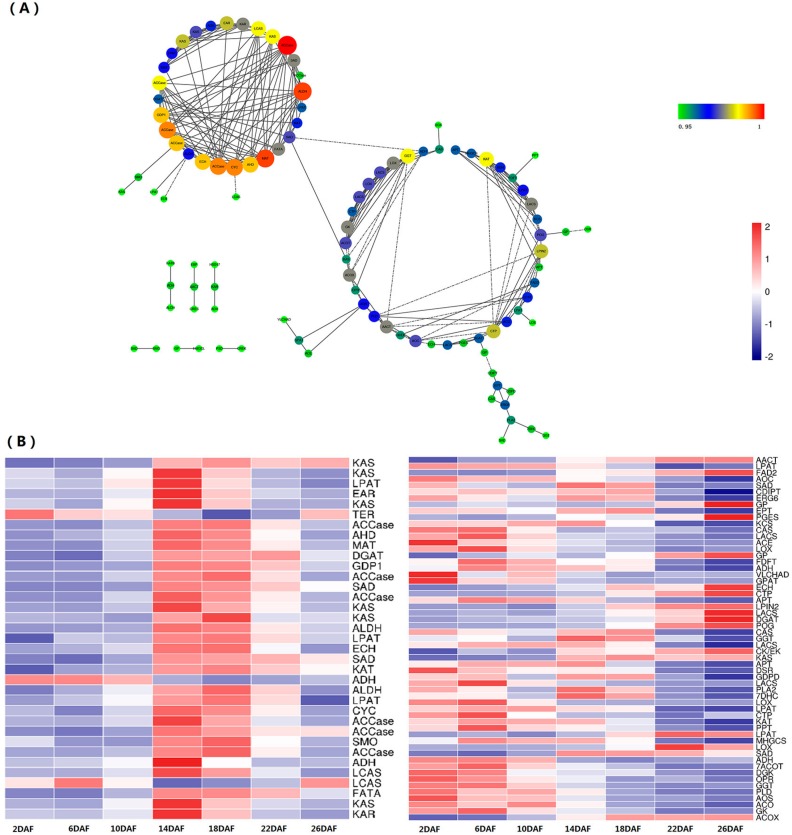
(**A**) A lipid metabolism-enriched module is presented with the degree-sorted circle layout of Cytoscape v3.4.10, with the sizes and colors of nodes reflecting the level of connectivity within the network. The bigger the node, the greater the number of connections it has. For clarity, the edges with correlation values smaller than 0.95 were removed; (**B**) Heat maps of the coexpression genes of lipid metabolism; The gene in left heat maps is correspond with the subnetwork I, and The genes in right heat maps is correspond with the subnetwork II. Each line represents a gene, and each column represents a specimen (stage). Depths of color in the red and blue rectangles indicate higher and lower represents the Z-score RNA expression lever.

Surveys and summaries of the expression patterns of these genes in the seeds at various developmental stages showed that subnetwork I genes were preferentially highly expressed in 14–18DAF, which showed consistent coexpressed tendency. Whereas subnetwork II guide genes showed not only consistent but also negative correlation coexpressed tendency. The de novo FA synthesis genes preferentially highly expressed in the early stages of development, and some transferase and peroxidase related lipid degradation or regulation preferentially highly expressed in the late stages of development (Figure 3).

### 2.6. Identification of Transcription Factor Families

**Figure 4:**
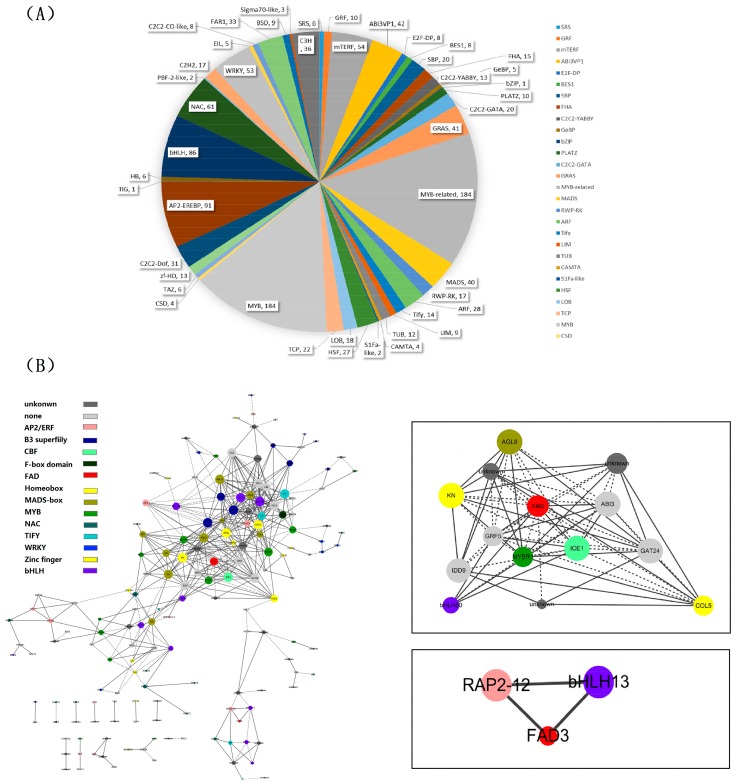
Transcription factor analysis. (**A**) Distribution of transcription factor (TF) families; (**B**) Coexpression network of transcript factors and fatty acid desaturase (FAD). The TF module was presented by Cytoscape v3.4.10. The rectangle indicates the TFs directly related to the FAD in the network. The solid line represents positive correlation, and the dotted line represents negative correlation.

For more information about the regulation of genes involved in lipid biosynthesis during the development of perilla seeds, the database PlnTFDB was used for identifying TFs in all unigenes. We identified a total of 1279 TFs, belonging to 45 TF families, among which the top five families were related to MYB (184), MYB-related (184), AP2-EREBP(91), bHLH(86), and NAC(61) (Figure 4A).

### 2.7. Identification and Functional Annotation of the Genes Coexpressing with Perilla Fatty Acid Desaturase Genes

ALA is abundantly accumulated in mature perilla seeds. It is important for health, but is scarce in our daily meals. For identifying the TFs involved in ALA biosynthesis and its regulation, the omega-3 and omega-6 fatty acid desaturase genes (DN26137_c0_g1 and DN25736_c0_g1) were defined as the “guide genes”. Based on these guide genes, a total of 162 TFs related to the two FADs (*r* > 0.95) were identified (Figure 4B and Table S3).

Most of these TFs belonged to six gene families, including MYB (16), AP2/ERF (12), bHLH (11), MADS-box (10), Zinc finger (10), and NAC (10) (Table S3). The *RAP2-12* (DN23390_c0_g1, *r* = 0.981) and *bHLH13* (DN35936_c1_g1, *r* = 0.962) TFs were found to be significantly correlated with omega-3 fatty acid desaturase. Thirteen TFs, including *ABI3* (DN33484_c3_g1, *r* = 0.986), *ICE1* (DN34648_c0_g1, *r* = 0.9877491), *IDD5* (DN33027_c2_g5, *r* = −0.965), *GRF5* (DN29710_c0_g1, *r* = 0.995), *KN* (DN24707_c1_g1_i1, *r* = −0.964), *COL5* (DN28351_c0_g1, *r* = −0.974), *GAT24* (DN30716_c0_g1, *r* = −0.963), *bHLH80* (DN31044_c0_g1, *r* = 0.982), *MYBR1* (DN27387_c0_g1, *r* = −0.989), *AGL8* (DN20914_c0_g1, *r* = −0.962) and three unknown genes, were significantly correlated with omega-6 fatty acid desaturase gene. Moreover, we identified the *WRI1* gene in the coexpression network.

### 2.8. The qRT-PCR Analysis of the Lipid Synthesis Genes in the Perilla

To confirm the gene expression results in our research, seven genes related to lipid synthesis and its regulation were selected for qRT-PCR analysis. The qRT-PCR results significantly correlated with RNA-Seq result were verified (*r* = 0.697) (Figure S4). Among them, *FAD3* (DN26137_c0_g1), *FAD2* (DN25736_c0_g1), *RAP2-12* (DN23390_c0_g1), *bHLH13* (DN35936_c1_g1), *WRI1*(DN28398_c0_g1), and *GNA1*(DN19692_c12_g4) showed highly expression levels in 18DAF; but *GRF5*(DN29710_c0_g1), gene were mainly expressed in 26DAF (Figure S5). Further, the remarkable correlation were verified between *FAD3* and *RAP2-12* (*r* = 0.975) and *bHLH13* (*r* = 0.979) in qRT-PCR analysis, and the other genes also showed remarkable correlation relationship with FAD3 gene (Table S4). These candidate TFs identified provided clues to clone and reveal the key molecular mechanisms underlying unsaturated FA biosynthesis and its regulation in perilla seeds.

## 3. Discussion and Conclusions

The transcriptome of perilla seed have been reported by Kim et al. [21], involving in four developmental stages in perilla seeds, and the genes related to acyl-lipid metabolism and FA and TAG synthesis were analysis. On the basis of research, we reported the RNA sequencing of seven different development phases in perilla seed. The number of unigenes and N50 slightly higher than previously reported [21] and support that homology analysis and identification of acyl-lipid genes. In total, we obtained 64,156 unigenes and 11,693 differentially expressed unigenes from our seed samples. The 8654, 10,904, and 22,263 unigenes with the COG, GO, and KEGG annotation, respectively were identified (Table 3). Among them, 150 unigenes were annotated as FA biosynthesis and TAG assembly genes (Figure 2 and Table S2). In the acyl-lipid genes, *PDAT* gene showed the relatively higher expression in perilla developed seeds that suggested it might be a priority pathway for the synthesis of perilla oil (Figure 3B). The TAG molecules can be stored in the form of an oil body (OB) surrounded by a layer of phospholipid membrane and embedded with several molecules of a protein called oleosin in mature plant seeds [35,36,37]. The main function of oleosin is to help stabilize OBs and prevent the fusion of OBs [38]. Five unigenes encoding oleosin were identified in the perilla seed transcriptome. It is noteworthy that three unigenes (DN25414_c0_g1, DN24171_c0_g1, and DN18941_c0_g1) showed high-level transcription in the late development, implying that they were involved in the formation of oil bodies. The analysis and identification of acyl-lipid genes effective supported the previous results [21,39]. Next, we analyzed the coexpression of lipid metabolism genes and identification of TFs. In our study, gene coexpression networks were identified from lipid biosynthesis genes of perilla. Two subnetworks, mainly representing the TAG biosynthesis and de novo FA synthesis genes, were obtained (Figure 3A). The results are similar with the transcriptome of *Arabidopsis* seeds from the globular to mature embryo stage, which reported three coexpression subnetworks involved in FA biosynthesis, oleosins and seed storage proteins, and TAG assembly pathways [40]. In perilla seed, the expression patterns of these genes also showed a coincident trend that the subnetwork I genes were preferentially highly expressed in the middle stage and the subnetwork II genes were preferentially highly expressed in the early or late stages of development (Figure 3B).

The perilla seed transcriptome and acyl-lipid metabolism genes have been systematically analyzed according to Kim et al. [21]. The *FAD2* and *FAD3* were showed obviously highly expressed in interim development phase of perilla seed [21]. As is known, the omega-6 (FAD2) and omega-3 (FAD3) fatty acid desaturase are important enzymes that catalyze the formation of ALA. In our study, *FAD2* and *FAD3* also showed some expression tendency, which provides key clues of the high ALA accumulation in perilla seeds. Therefore, we used the two *FAD*s as inquiry to execute the co-expression analysis, and found 162 TFs related to these two *FADs*. Acquired TFs belonged to important regulatory gene families of lipid biosynthesis and seed development, such as AP2/ERF (12), bHLH (11), MADS-box (10), B3 (9), MYB (16), Zinc finger (10), and so on (Figure 4A). As reported previously, the AP2/ERF-family TFs are involved in the control of primary and secondary metabolism, growth and development, and responses to environmental stimuli [41]. The *WRI* gene, encoding a 48.4-kDa protein with two AP2 binding domains, directly regulates oil accumulation and storage in seeds, leading to the production of enough oil needed for seedling establishment [42]. The main target genes of *WRI1* are involved in glycolysis and FA synthesis in *Arabidopsis* [43]. In perilla FAD coexpression results, *WRI1* and three other AP2/ERF family members (*ERF*, *AIL*, and *RAP*) were identified. The basic helix-loop-helix (bHLH) proteins are a large superfamily of TFs. bHLH TF plays a key role in the transcriptional regulation of genes related to storage lipid biosynthesis and accumulation during seed development [44]. The bHLH proteins were associated with fruit development and ripening in tomato recently report by Sun et al. [45]. More bHLH and bHLH-related genes were identified is a key regulator of *FAD2* expression in *Arabidopsis*, sesame and cotton [44,46,47]. The SebHLH protein mediates transactivation of the SeFAD2 gene promoter through binding to E- and G-box elements [44]. The *bHLH* (*r* = 0.962) showed coexpression with perilla *FAD3* implies the potential function of ALA biosynthesis and its regulation (Figure 4B). Next, growth-regulating factor (GRF4) is an important TF, which was reported to regulate grain size and yield by OsmiR396 controls in rice [48]. It was found to coexpress with two FADs. This TF gene from coexpression analysis gave more clue for future research to uncover ALA biosynthesis and its regulation mechanism. The possible TFs regulating ALA biosynthesis and co-expression research showed more information to understand ALA underlying biosynthesis and regulation than previous reports [21].

In conclusion, we focused on the key genes and TFs involved in ALA biosynthesis in perilla seed. The results will serve as an important basis for perilla seed development analysis and provide more critical information and characterization of ALA synthesis mechanism and TAG biosynthesis in oilseeds.

## 4. Materials and Methods

### 4.1. Plant Materials, Library Construction, and Transcriptome Sequencing

The plants of *Pf.* var. *frutescens* were grown in a farm of the Guizhou Rapeseed Institute, Guizhou Province, China (31°39′ N, 119°19′ E). For transcriptome samples, the seeds at seven development stages, that is, 2, 6, 10, 14, 18, 22, and 26 days after flowering (DAF), were collected from the same individual plant and were quickly frozen in liquid nitrogen. The total RNA from all seed samples was extracted separately using the RNAprep Pure Plant Kit (Tiangen, Beijing, China) following the manufacturer’s instructions. An amount of 1–2 µg total RNA per sample with 28S/18S RNA ratio ≥ 1.8 used for library preparation. The mRNA sequencing library was constructed using the NEBNext^®^ Ultra RNA Library Prep Kit (New England Biolabs Inc., Ipswich, MA, USA). The sequencing library was analyzed using the Agilent 2100 Bioanalyzer with a minimum integrity number (RIN) value of 7 and the insertion element are 250–300 bp. The library was sequenced using an Illumina HiSeq™ 2000 sequencing system (Illumina Inc., San Diego, CA, USA).

### 4.2. Data Filtering and De Novo Assembly

After removing the adapter sequences, low-quality sequences with N percentage (i.e., the percentage of nucleotides in read which could not be sequenced) over 5%, and those containing more than 50% bases with *q*-value ≤ 5, and sequences shorter than 35 bases, the clean reads from seven different developmental-phase seeds were obtained. The clean reads were de novo assembled into transcripts using the Trinity software with min_kmer_cov set to 4 and all other parameters set to default values [49]. The complete open reading frames (ORFs) of unigenes were predicted using the program TransDecoder (https://transdecoder.github.io/).

### 4.3. Functional Annotation of Unigenes

For functional annotation, all assembled unigenes were searched against the non-redundant protein database of the National Center for Biotechnology Information (NCBI) (NR, http://www.ncbi.nlm.nih.gov), Swiss-Prot (http://www.expasy.ch/sprot), TrEMBL (http://www.ebi.ac.uk/trembl), KOG (http://www.ncbi.nlm.nih.gov/COG/) and the Kyoto Encyclopedia of Genes and Genomes (KEGG, http://www.genome.jp/kegg) using the basic local alignment search tool (BLAST) program with a cut-off *e*-value of <1e^−5^. Subsequently, gene ontology (GO) annotation was performed using the Blast2GO and ClusterProfiler [50,51] programs to determine the GO functional classifications according to molecular function, biological process, and cellular component.4.4. Identification and Coexpression Analysis of Lipid Metabolism-Related Genes.

The genes related to lipid metabolism were selected according to the KEGG annotation results. For coexpression analysis, the Pearson correlation coefficient (r) based on FPKM was calculated using Python [52]. Coexpression networks were analyzed across all developmental stages. The genes that were not expressed in 60% of the samples were excluded from the analysis. The paired genes with a Pearson correlation coefficient (r) greater than 0.95 were considered as significantly coexpressed genes and were selected to build a coexpression network using the Perl script, the Perl script available at https://git.coding.net/zhangtianyuan/coexpression-analysis.git.

### 4.4. Identification of Differentially Expressed Genes

The abundance of unigenes was normalized using the FPKM (Fragments Per Kilobase of exon per Million mapped fragments) values. The differential gene expression analysis was performed on the paired samples of the seven developmental stages of perilla seeds using the edgeR package [53]. The absolute value of log_2_Foldchange > 1 and the false discovery rate (FDR) <0.05 was used to identify the significance of different gene expression. Subsequently, the GO functional enrichment and KEGG pathway enrichment analyses of the DEGs were performed using the software tools GOseq and KOBAS, respectively [54,55].

### 4.5. Transcription Factor Identification and Co-Expression with Fatty Acid Desaturase

Transcription factor (TF) families were identified using the known plant TF database PlnTFDB (http://plntfdb.bio.uni-potsdam.de/v3.0). To select the possible TFs regulating ALA biosynthesis, two highly expressed fatty acid desaturase genes—*FAD3* (catalyzes linoleic acid to α-linolenic acid) and *FAD2* (catalyzes oleic acid to linoleic acid)—were constructed for a co-expressed analysis with a default value 0.6 [51]. As described above, the paired genes showing a Pearson correlation coefficient (r) greater than 0.95 were considered as significantly coexpressed genes and selected to build a coexpression network using the Perl script. Data correlation and visualization were performed using the program Cytoscape v3.4.10 [56].

### 4.6. Quantitative Real-Time PCR Analysis

The selected genes were confirmed by qRT-PCR using Rotor-GeneQ (Qiagen, Hilden, Germany) with SYBR Green qPCR SuperMix (Transgene, Beijing, China). Plant materials were planted and collected in same condition as transcriptome sample, RNA were extracted and transcripted to cDNA as sample template. The gene primers were designed using primer premier 3.0. The qPCR using two-step method, and the genes expression quantity were analyzed by 2^−ΔΔ*C*t^ method using the perilla actin sequence as the internal gene [57].We all used the four stages (2, 10, 18, and 26 DAF) in samples from four biologic repetition by qRT-PCR.

The qPCR reactions as followed: Each 20 μL reaction mixture contained 10 μL of SYBR Green qPCR SuperMixUDG TaqTM, 1.5 μL of diluted cDNA, 0.4 μL of each primer (10 μM), 0.4 μL of ROX Reference Dye (50*) and 7.7 μL of double distilled water. The qPCR cycling conditions were as follows: 50 °C for 2min; followed by 40 cycles of 94 °C for 5 s, and 60 °C 30 s in PCR strip tubes.

## Figures and Tables

**Table 1:** Summary of perilla seed transcriptome data sequenced by the Illumina platform.

Sample ID	Total Reads	Total Bases	GC Content	Q20	Q30
2DAF	18,094,914	2,261,864,250	46.64%	94.64%	89.76%
6DAF	17,885,482	2,235,685,250	47.88%	94.36%	89.70%
10DAF	17,075,076	2,134,384,500	49.31%	94.38%	89.77%
14DAF	16,747,764	2,093,470,500	49.61%	94.25%	89.58%
18DAF	14,929,122	1,866,140,250	51.42%	93.70%	88.05%
22DAF	20,751,538	2,593,942,250	52.52%	93.74%	88.71%
26DAF	21,517,792	2,689,724,000	47.89%	95.39%	91.21%
Total	127,001,688	15,875,211,000			

DAF: days after flowering.

**Table 2:** Statistics of de novo assembly of sequence reads.

Item	Total Number (bp)	N50 (bp)	Median Length (bp)	Average Length (bp)	Total Length (bp)
Transcripts	104,638	1608	600	968	101,378,085
Unigenes	64,156	1417	402	777	49,883,108

**Table 3:** Statistics of annotations of assembled unigenes.

Database	Account	Percentage ^c^
NR ^a^	32,132	50.08%
KEGG classified unigenes	10,904	17.00%
COG classified unigenes	8654	14.47%
GO classified unigenes	22,263	34.70%
Blast_hit ^b^	31,287	48.77%
Pfam classified unigenes	19,340	30.15%
Eggnog classified unigenes	9425	14.69%
TmHMM classified unigenes	6719	10.47%
SignalP classified unigenes	2354	3.67%
All annotated unigenes	39,760	61.97%
All	64,156	100.00%

^a^ NCBI non-redundant database; ^b^ SWISSPORT and TREMBLE database; ^c^ Percentage of all assembled unigenes.

**Table 4:** Differentially Expressed Genes (DEGs) between two different developmental stages.

	2DAF	6DAF	10DAF	14DAF	18DAF	22DAF	26DAF
2DAF	0						
6DAF	137↑ 263↓	0					
10DAF	876↑ 1316↓	294↑ 558↓	0				
14DAF	1755↑ 1399↓	1352↑ 1227↓	353↑ 368↓	0			
18DAF	3022↑ 1813↓	2370↑ 1583↓	2001↑ 955↓	257↑ 85↓	0		
22DAF	3666↑ 2031↓	3188↑ 1816↓	2941↑ 1363↓	1350↑ 674↓	47↑ 83↓	0	
26DAF	4751↑ 2221↓	4730↑ 2119↓	4445↑ 1692↓	2615↑ 1268↓	1253↑ 949↓	360↑ 122↓	0

DAF: days after flowering.

## Supplementary Materials

Supplementary materials can be found at www.mdpi.com/1422-0067/18/11/2433/s1.
