# Supplementary material for: RNA Sequencing and Coexpression Analysis Reveal Key Genes Involved in α-Linolenic Acid Biosynthesis in Perilla frutescens Seed
Source: Int J Mol Sci. 2017 Nov 16;18(11):2433. doi: 10.3390/ijms18112433 (PMC5713401; doi:10.3390/ijms18112433)
Supplement: Supplementary file 1 [file ijms-18-02433-s001.zip › ijms-241170-suppl/Legends of Suppl Mat.pdf]

Figure S1. The COG annotation for all assembled unigenes. Of 8654 unigenes were classified into 25 COG categories.

Figure S2. Gene ontology classification of the perilla transcriptome. Gene ontology (GO) terms summarized into three main GO categories (biological process, cellular component, and molecular function).

Figure S3. Functional classification and pathway assignment of assembled unigenes by KEGG.

Figure S4. Correlations of expression levels by real time PCR (log2qPCR, x axis) with data obtained using the log2 RNA-Seq platform (y-axis) for different genes and time points. One point represents a sample, and seven genes are analyzed through qRT-PCR. The name of TF has been marked in legend.

Figure S5. Relative expression of selected unigenes in developing seeds.

Table S1. Number of lipid metabolism gene

Table S2. Enzymesprotein related to FA biosynthesis and metabolism identified by annotation of the Perilla unigenes

Table S3. List of co-expressed transcript factor gene.

Table S4. The Pearson correlation analysis between FAD3 and target genes.
