# Supplementary material for: RNA Sequencing and Coexpression Analysis Reveal Key Genes Involved in α-Linolenic Acid Biosynthesis in Perilla frutescens Seed
Source: Int J Mol Sci. 2017 Nov 16;18(11):2433. doi: 10.3390/ijms18112433 (PMC5713401; doi:10.3390/ijms18112433)
Supplement: Supplementary file 1 [file ijms-18-02433-s001.zip › ijms-241170-suppl/References of Suppl Mat.pdf]

58. Licausi, F.; Ohme-Takagi, M.; Perata, P. APETALA2/Ethylene Responsive Factor (AP2/ERF) transcription factors: Mediators of stress responses and developmental programs. *New Phytol*. **2013**, *199*, 639, doi:10.1111/nph.12291.
59. Hinz, M.; Wilson, I.W.; Yang, J.; Buerstenbinder, K.; Llewellyn, D.; Dennis, E.S.; Dolferus, R. Arabidopsis RAP2.2: An Ethylene Response Transcription Factor That Is Important for Hypoxia Survival. *Plant Physiol*. **2010**, *153*, 757, doi:10.1104/pp.110.155077.
60. Mantegazza, O.; Gregis, V.; Mendes, M.A.; Morandini, P.; Alvesferreira, M.; Patreze, C.M.; Colombo, L. Analysis of the arabidopsis REM gene family predicts functions during flower development. *Ann. Bot*. **2014**, *114*, 1507–1515, doi:10.1093/aob/mcu124.
61. Jia, H.; Suzuki, M.; Mccarty, D.R. Regulation of the seed to seedling developmental phase transition by the LAFL and VAL transcription factor networks. *Wiley Interdiscip. Rev. Dev. Biol*. **2014**, *3*, 135–145, doi:10.1002/wdev.126.
62. Chandler, J.W. Auxin response factors. *Plant Cell Environ*. **2015**, *39*, 1014–28, doi:10.1111/pce.12662.
63. Payne, C.T.; Zhang, F.; Lloyd, A.M. GL3 encodes a bHLH protein that regulates trichome development in arabidopsis through interaction with GL1 and TTG1. *Genetics* **2000**, *156*, 1349–1362.
64. Wang, L.; Ying, Y.; Narsai, R.; Ye, L.; Zheng, L.; Tian, J.; Whelan, J.; Shou, H. Identification of OsbHLH133 as a regulator of iron distribution between roots and shoots in Oryza sativa. *Plant Cell Environ*. **2013**, *36*, 224–236, doi:10.1111/j.1365-3040.2012.02569.x.
65. Fernández-Calvo, P.; Solano, R. The Arabidopsis bHLH transcription factors MYC3 and MYC4 are targets of JAZ repressors and act additively with MYC2 in the activation of jasmonate responses. *Plant Cell* **2011**, *23*, 701–715, doi:10.1105/tpc.110.080788.
66. Broholm, S.K.; Tähtiharju, S.; Laitinen, R.A.E.; Albert, V.A.; Teeri, T.H.; Elomaa, P. A TCP domain transcription factor controls flower type specification along the radial axis of the Gerbera (Asteraceae) inflorescence. *Proc. Natl. Acad. Sci. USA* **2008**, *105*, 9117–9122, doi:10.1073/pnas.0801359105.
67. Chinnusamy, V.; Ohta, M.; Kanrar, S.; Lee, B.; Hong, X.; Agarwal, M.; Zhu, J.K. ICE1: A regulator of cold-induced transcriptome and freezing tolerance in Arabidopsis. *Genes Dev*. **2003**, *17*, 1043, doi:10.1101/gad.1077503.
68. Lai, C.P.; Lee, C.L.; Chen, P.H.; Wu, S.H.; Yang, C.C.; Shaw, J.F. Molecular analyses of the Arabidopsis TUBBY-like protein gene family. *Plant Physiol*. **2004**, *134*, 1586–1597, doi:10.1104/pp.103.037820.
69. Reiser, L.; Sánchez-Baracaldo, P.; Hake, S. Knots in the family tree: Evolutionary relationships and functions of knox homeobox genes. *Plant Mol. Biol*. **2000**, *42*, 151, doi:10.1007/978-94-011-4221-2_7
70. Wang, W.; Peng, W.; Ying, L.; Hou, X.L. Genome-wide analysis and expression patterns of ZF-HD transcription factors under different developmental tissues and abiotic stresses in Chinese cabbage. *Mol. Genet. Genom*. **2016**, *291*, 1451–1464, doi:10.1007/s00438-015-1136-1.
71. Lee, W.K.; Yun, J.H.; Lee, W.; Cho, M.H. DNA-Binding Domain of AtTRB2 Reveals Unique Features of a Single Myb Histone Protein Family that Binds to Both Arabidopsis-and Human-Type Telomeric DNA Sequences. *Mol. Plant* **2012**, *5*, 1406–1408, doi:10.1093/mp/sss063.
72. Yanofsky, M.F.; Ma, H.; Bowman, J.L.; Drews, G.N.; Feldmann, K.A.; Meyerowitz, E.M. Protein encoded by the Arabidopsis homeotic gene agamous resembles transciption factors. *Nature* **1990**, doi:10.1038/346035a0.
73. Wang, H.; Caruso, L.V.; Downie, A.B.; Perry, S.E. The embryo MADS domain protein AGAMOUS-Like 15 directly regulates expression of a gene encoding an enzyme involved in gibberellin metabolism. *Plant Cell* **2004**, *16*, 1206–1219, doi:10.1105/tpc.021261.
74. Kobayashi, K.; Yasuno, N.; Sato, Y.; Yoda, M.; Yamazaki, R.; Kimizu, M.; Kyozuka, J. Inflorescence Meristem Identity in Rice Is Specified by Overlapping Functions of Three AP1/FUL-Like MADS Box Genes and PAP2, a SEPALLATA MADS Box Gene. *Plant Cell* **2012**, *24*, 1848–1859, doi:10.1105/tpc.112.097105.
75. Jetha, K.; Theißen, G.; Melzer, R. Arabidopsis SEPALLATA proteins differ in cooperative DNA-binding during the formation of floral quartet-like complexes. *Nucleic Acids Res*. **2011**, *42*, 10927–10942, doi:10.1093/nar/gku755.
76. Galego, L.; Almeida, J. Role of DIVARICATA in the control of dorsoventral asymmetry in Antirrhinum flowers. *Genes Dev.* **2002**, *16*, 880, doi:10.1101/gad.221002.
77. Gocal, G.; Sheldon, C.C.; Gubler, F.; Moritz, T.; Bagnall, D.J.; Macmillan, C.P.; King, R.W. GAMYB-like Genes, Flowering, and Gibberellin Signaling in Arabidopsis. *Plant Physiol*. **2001**, *127*, 1682–1693, doi:10.1104/pp.010442.
78. Schaffer, R.; Ramsay, N.; Samach, A.; Corden, S.; Putterill, J.; Carré, I.A.; Coupland, G. The late elongated hypocotyl mutation of arabidopsis disrupts circadian rhythms and the photoperiodic control of flowering. *Cell* **1998**, *93*, 1219, doi:10.1016/S0092-8674(00)81465-8.
79. Chen, Y.; Yang, X.; He, K.; Liu, M.; Li, J.; Gao, Z.; Lin, Z.; Zhang, Y.; Liu, Z.; Zhang, Y.; et al. The MYB transcription factor superfamily of Arabidopsis: Expression analysis and phylogenetic comparison with the rice MYB family. *Plant Mol. Biol*. **2006**, *60*, 107–124, doi:10.1007/s11103-005-2910-y.
80. Ooka, H.; Satoh, K.; DOI, K.; Nagata, T.; Otomo, Y.; Murakami, K.; Matsubara, K.; Osato, N.; Kawai, J.; Carninci, P.; et al. Comprehensive analysis of nac family genes in oryza sativa and arabidopsis thaliana. *DNA Res.* **2003**, *10*, 239, doi:10.1093/dnares/10.6.239.
81. Ye, H.; Du, H.; Tang, N.; Li, X.; Xiong, L. Identification and expression profiling analysis of TIFY family genes involved in stress and phytohormone responses in rice. *Plant Mol. Biol*. **2009**, *71*, 291–305, doi:10.1007/s11103-009-9524-8.

82. Wu, K.; Guo, Z.; Wang, H.; Li, J. The WRKY Family of Transcription Factors in Rice and Arabidopsis and Their Origins. *DNA Res*. **2005**, *12*, 9–26, doi:10.1093/dnares/12.1.9.
83. Wang, D.; Guo, Y.; Wu, C.; Yang, G.; Li, Y.; Zheng, C. Genome-wide analysis of CCCH zinc finger family in Arabidopsis and rice. *BMC Genom*. 2008, *9*, 44, doi:10.1186/1471-2164-9-44.
84. Wong, A.C.S.; Hecht, V.F.G.; Picard, K.; Diwadkar, P.; Laurie, R.E.; Wen, J.; Weller, J.L. Isolation and functional analysis of CONSTANS-LIKE genes suggests that a central role for CONSTANS in flowering time control is not evolutionarily conserved in Medicago truncatula. *Front. Plant Sci*. **2014**, *5*, 486–486, doi:10.3389/fpls.2014.00486.
85. Liu, X.; Zhang, H.; Zhao, Y.; Feng, Z.; Li, Q.; Yang, H.Q.; He, Z.H. Auxin controls seed dormancy through stimulation of abscisic acid signaling by inducing ARF-mediated ABI3 activation in Arabidopsis. *Proc. Natl. Acad. Sci. USA* **2013**, *110*, 15485, doi:10.1073/pnas.1304651110.
86. Galon, Y.; Nave, R.; Boyce, J.M.; Nachmias, D.; Knight, M.R.; Fromm, H. Calmodulin-binding transcription activator (CAMTA) 3 mediates biotic defense responses in Arabidopsis. *FEBS Lett.* **2008**, *582*, 943, doi:10.1016/j.febslet.2008.02.037.
87. Pietrzykowska, M.; Suorsa, M.; Semchonok, D.A.; Tikkanen, M.; Boekema, E.J.; Aro, E.M.; Jansson, S. The light-harvesting chlorophyll a/b binding proteins Lhcb1 and Lhcb2 play complementary roles during state transitions in Arabidopsis. *Plant Cell* **2015**, *26*, 3646–3660, doi:10.1105/tpc.114.127373.
88. Imai, R.; Kim, M.H.; Sasaki, K.; Sato, S.; Sonoda, Y. Cold Shock Domain Proteins in Arabidopsis: Functions in Stress Tolerance and Development. In *Plant and Microbe Adaptations to Cold in a Changing World*; Springer: New York, NY, USA 2013; pp. 131–142, doi:10.1007/978-1-4614-8253-6_11.
89. Yoshida, H.; Hirano, K.; Sato, T.; Mitsuda, N.; Nomoto, M.; Maeo, K.; Ishiguro, S. DELLA protein functions as a transcriptional activator through the DNA binding of the indeterminate domain family proteins. *Proc. Natl. Acad. Sci. USA* **2014**, 111, 7861, doi:10.1073/pnas.1321669111.
90. Feng, B.; Ma, S.; Chen, S.; Ning, Z.; Zhang, S.; Yu, B.; Yu, Y.; Le, B.; Chen, X.; Dinesh-Kumar, S.P.; et al. Parylation of the forkhead-associated domain protein dawdle regulates plant immunity. *EMBO Rep.* **2016**, *17*, 1799, doi:10.15252/embr.201642486.
91. Bi, Y.M.; Zhang, Y.; Signorelli, T.; Zhao, R.; Zhu, T.; Rothstein, S. Genetic analysis of Arabidopsis GATA transcription factor gene family reveals a nitrate-inducible member important for chlorophyll synthesis and glucose sensitivity. *Plant J. Cell Mol. Biol*. **2005**, *44*, 680–692, doi:10.1111/j.1365-313X.2005.02568.x.
92. Von, K.P; Scharf, K.D; Nover, L. The diversity of plant heat stress transcription factors. *Trends Plant Sci.* **2007**, *12*, 452, doi:10.1016/j.tplants.2007.08.014.
93. Seo, P.J.; Ryu, J.; Kang, S.K.; Park, C.M. Modulation of sugar metabolism by an INDETERMINATE DOMAIN transcription factor contributes to photoperiodic flowering in Arabidopsis. *Plant J. Cell Mol. Biol.* **2011**, *65*, 418–429, doi:10.1111/j.1365-313X.2010.04432.x.
94. Kerstetter, R.A.; Bollman, K.; Taylor, R.A.; Bomblies, K.; Poethig, R.S. KANADI regulates organ polarity in Arabidopsis. *Nature* **2011**, *411*, 706–709, doi:10.1038/35079629.
95. Arnaud, D.; Dejardin, A.; Leple, J.; Lesagedescauses, M.; Pilate, G. Genome-Wide Analysis of LIM Gene Family in Populus trichocarpa, Arabidopsis thaliana, and Oryza sativa. *DNA Res.* **2007**, *14*, 103–116, doi:10.1093/dnares/dsm013.
96. Rouached, H.; Secco, D.; Arpat, B.; Poirier, Y. The transcription factor phr1 plays a key role in the regulation of sulfate shoot-to-root flux upon phosphate starvation in arabidopsis. *BMC Plant Biol.* **2011**, *11*, 19, doi:10.1186/1471-2229-11-19.
97. Zhang, Z.; Ogawa, M.; Fleet, C.M.; Zentella, R.; Hu, J.; Heo, J.; Lim, J.; Kamiya, Y.; Yamaguchi, S.; Sun, T. Scarecrow-like 3 promotes gibberellin signaling by antagonizing master growth repressor DELLA in Arabidopsis. *Proc. Natl. Acad. Sci. USA* **2011**, *108*, 2160–2165, doi:10.1073/pnas.1012232108.
98. Buszewicz, D.; Archacki, R.; Palusiński, A.; Kotliński, M.; Fogtman, A.; Iwanicka-Nowicka, R.; Sosnowska, K.; Kuciński, J.; Pupel, P.; Olędzki, J.; et al. HD2C histone deacetylase and a SWI/SNF chromatin remodeling complex interact and both are involved in mediating the heat stress response in Arabidopsis. *Plant Cell Environ.* **2016**, *39*, 2108–2122, doi:10.1111/pce.12756.
99. Fulcher, N.; Riha, K. Using Centromere Mediated Genome Elimination to Elucidate the Functional Redundancy of Candidate Telomere Binding Proteins in Arabidopsis thaliana. *Front. Genet*. **2015**, *6*, 349, doi:10.3389/fgene.2015.00349.
100. Bowman, J.L. The YABBY gene family and abaxial cell fate. *Curr. Opin. Plant Biol*. **2000**, *3*, 17–22, doi:10.1016/S1369-5266(99)00035-7.
101. Merlot, S.; Gosti, F.; Guerrier, D.; Vavasseur, A.; Giraudat, J. The ABI1 and ABI2 protein phosphatases 2C act in a negative feedback regulatory loop of the abscisic acid signalling pathway. *Plant J*. **2001**, *25*, 295–303, doi:10.1046/j.1365-313x.2001.00965.x.
102. Chen, X.; Zhang, Z.; Liu, D.; Kai, Z.; Li, A.; Long, M. SQUAMOSA Promoter-Binding Protein-Like Transcription Factors: Star Players for Plant Growth and Development. *J. Integr. Plant Biol.* **2010**, *52*, 946–951, doi:10.1111/j.1744-7909.2010.00987.x.
