# Supplementary figures and images for: RNA Sequencing and Coexpression Analysis Reveal Key Genes Involved in α-Linolenic Acid Biosynthesis in Perilla frutescens Seed
Source: Int J Mol Sci. 2017 Nov 16;18(11):2433. doi: 10.3390/ijms18112433 (PMC5713401; doi:10.3390/ijms18112433)

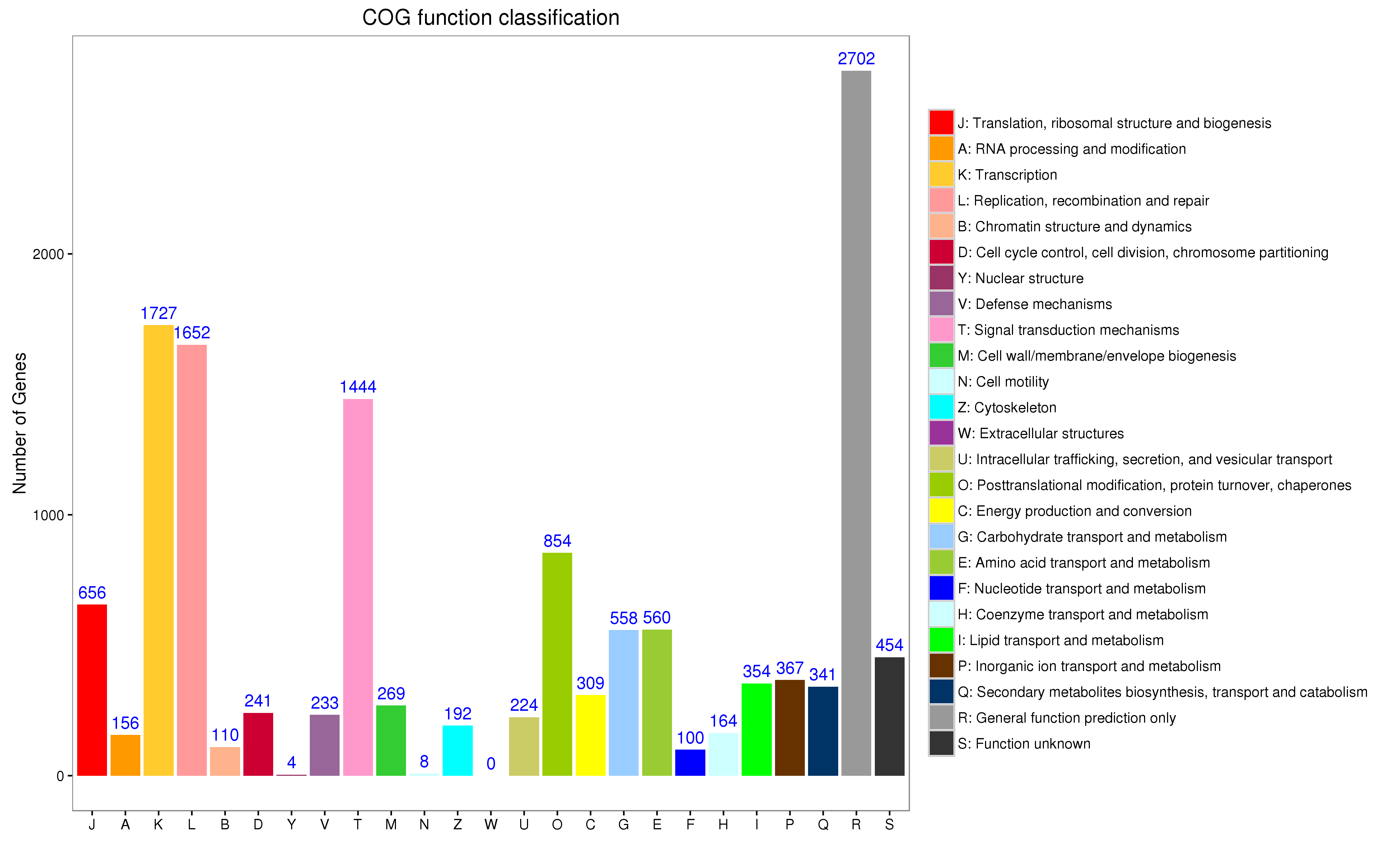

Supplement: Supplementary file 1 [file ijms-18-02433-s001.zip › ijms-241170-suppl/Figure S1. The COG annotation for all assembled unigenes. Of 8654 unigenes were classified into 25 COG.jpg]

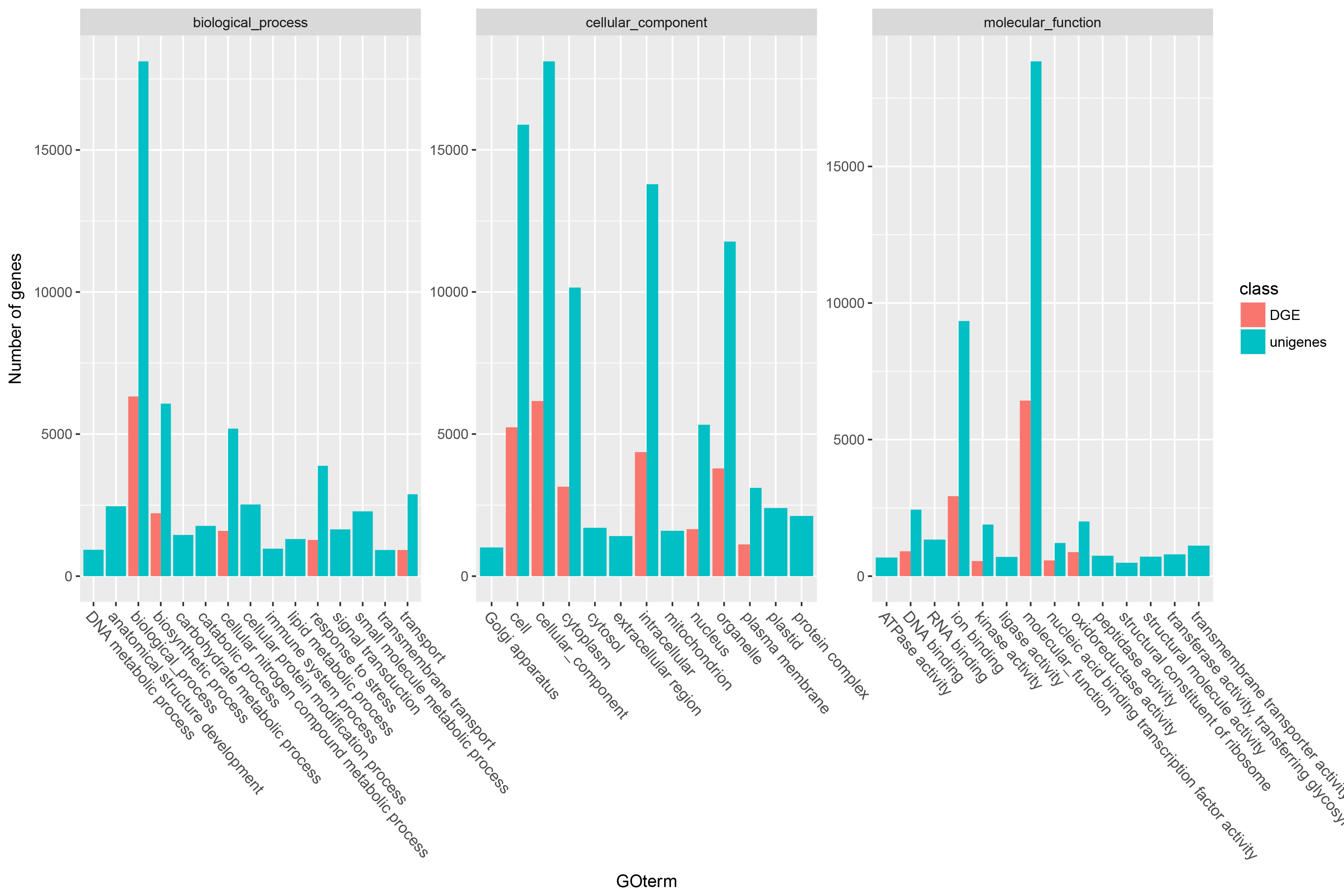

Supplement: Supplementary file 1 [file ijms-18-02433-s001.zip › ijms-241170-suppl/Figure S2. GO1.jpg]

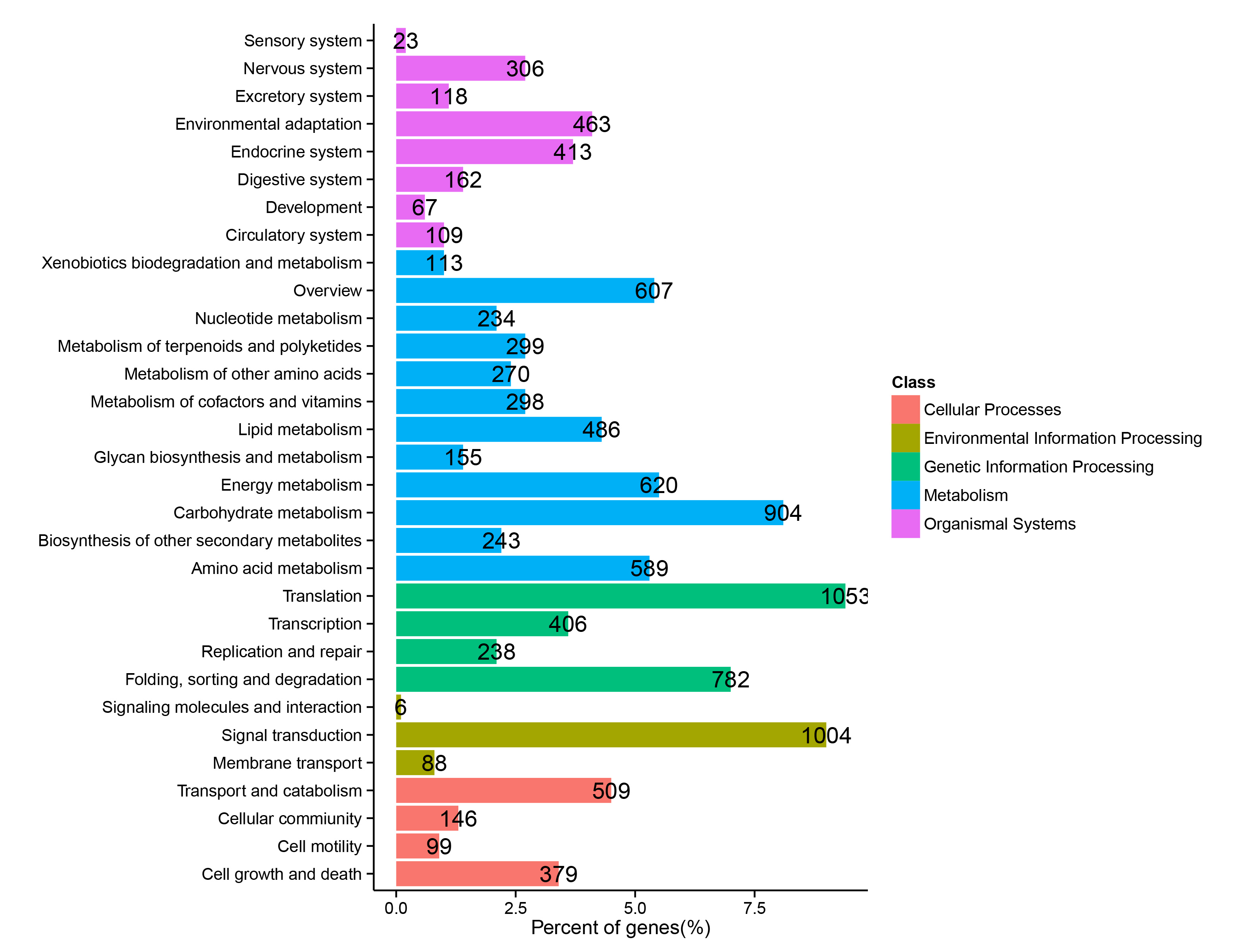

Supplement: Supplementary file 1 [file ijms-18-02433-s001.zip › ijms-241170-suppl/Figure S3. Functional classification and pathway assignment of assembled unigenes by KEGG..jpg]

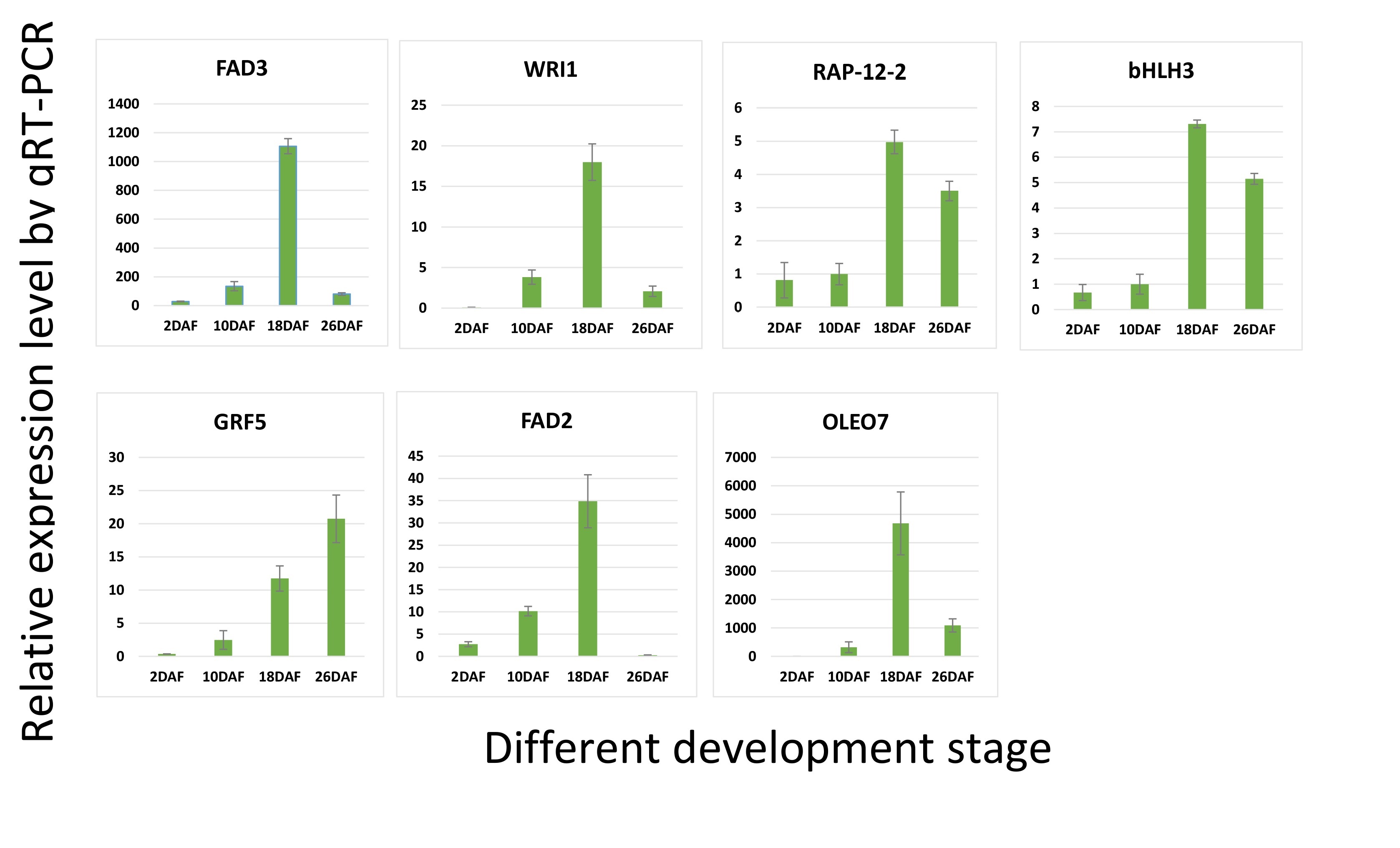

Supplement: Supplementary file 1 [file ijms-18-02433-s001.zip › ijms-241170-suppl/Figure S4. Relative expression of selected unigenes in developing seeds-3.jpg]

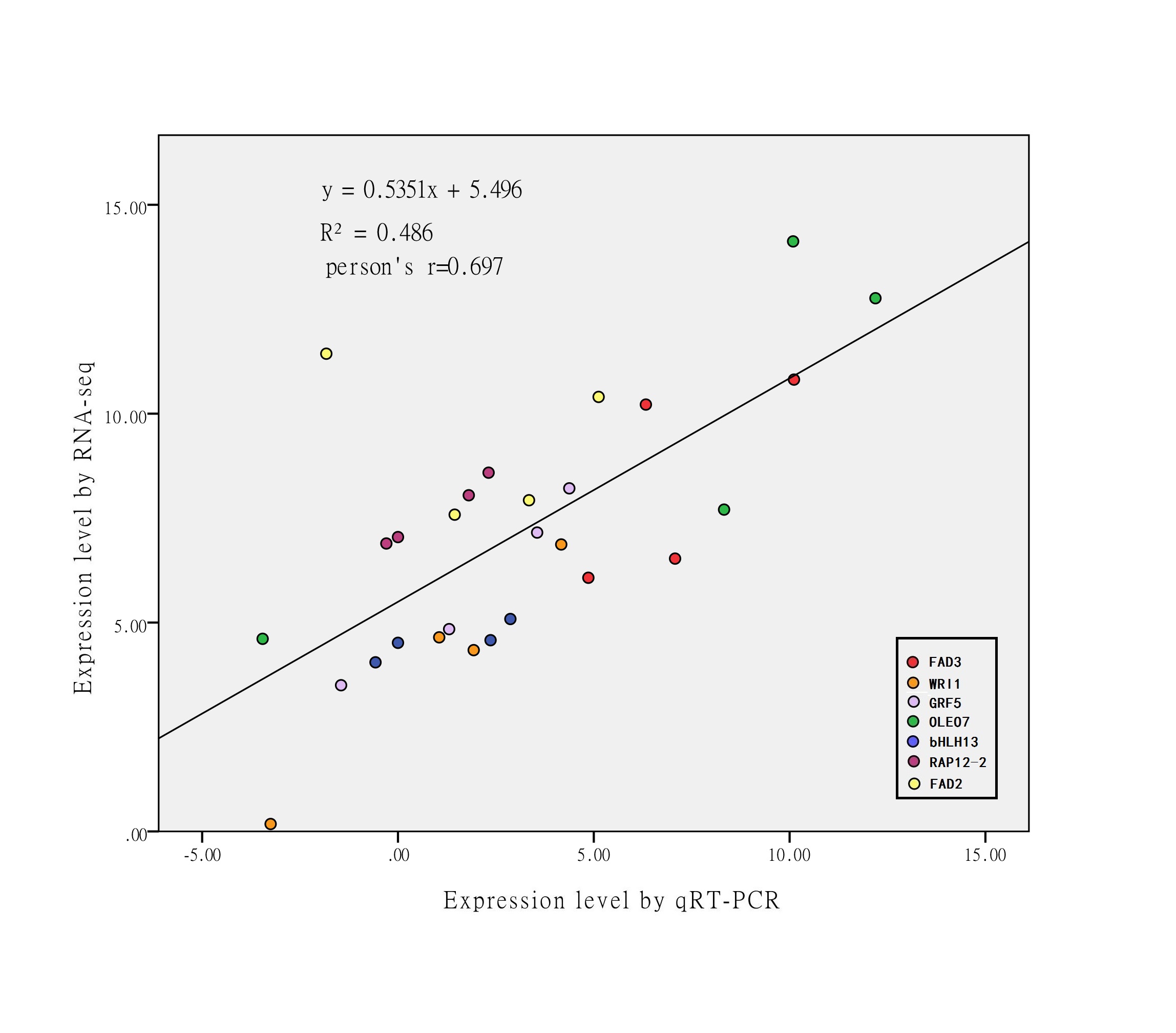

Supplement: Supplementary file 1 [file ijms-18-02433-s001.zip › ijms-241170-suppl/Figure S5. Correlations of expression levels.jpg]
